# Reply to: Endothelium-dependent and -independent functions in migraineurs

**DOI:** 10.1007/s10396-018-0910-1

**Published:** 2018-10-22

**Authors:** Kazumi Fujioka

**Affiliations:** Division of Laboratory Medicine, Department of Pathology and Microbiology, Nihon University School of Medicine, Tokyo, Japan

Reply from the authors: We thank Dr. Yetkin for his interest in our article titled “Increased nitroglycerin-mediated vasodilation in migraineurs without aura in the interictal period” [1]. Briefly, we found comparable values of flow-mediated vasodilation (FMD) between migraineurs and nonmigraineurs, but higher values of nitroglycerin-mediated vasodilation (NMD) in migraineurs than in nonmigraineurs. Our data indicated that endothelial dysfunction may not underlie the pathogenesis of increased NMD as previously reported [2, 3], and that the sensitive dilator response to NTG was due to a vascular smooth muscle cell (VSMC) abnormality in migraineurs without aura. Although increased dilator response to NTG has been reported in those with proven endothelial dysfunction in spastic coronary arteries in coronary spastic angina [4], Adams et al. reported that smooth muscle dysfunction occurred independently of impaired endothelium-dependent dilation in the brachial artery in adults at risk of atherosclerosis [5]. This response [4] might be a specific phenomenon in spastic coronary arteries in coronary spastic angina. To determine whether the decreased FMD specifically depends on endothelial dysfunction or whether it is derived from VSMC dysfunction or the change in vascular structures, endothelium-independent vasodilation should be assessed simultaneously using a direct smooth muscle relaxant such as NTG [6].

It has also been suggested that inflammation and/or oxidative stress result in reduction of the bioconversion of GTN to NO within VSMC, producing impaired NMD [7]. Like obese patients [7], impaired NMD has been demonstrated in patients with atherosclerosis. Increased NMD with normal FMD is a specific reaction. The sensitive dilator response to NTG is due to a VSMC abnormality in migraineurs without aura. Our increased NMD value agreed with that in Yetkin’s report [3], but it was different from that in a previous report [8]. We think that NO sensitivity to NTG in patients with migraine without aura is a specific and selective response, and that patients with migraine may have systemic NO sensitivity to NTG, as previously described [1].

Migraine has been shown to be linked with atherosclerosis associated with endothelial dysfunction. Our data did not indicate endothelial dysfunction, including vascular reactivity, FMD and biomarker, von Willebrand factor (vWF) [1]. Additionally, there were no differences in FMD between migraine patients with or without aura and controls in studies using strict criteria [9, 10].

Furthermore, normal endothelial function with increased arterial stiffness caused primarily by structure changes has been demonstrated using a novel noninvasive peripheral finger plethysmograph (EndoPAT) [11]. These controversial results might be explained by differences in age, gender, method of analysis, and adjustments for confounding factors.

Genetically, it has been reported that vascular dysfunction and possibly also smooth muscle dysfunction likely have roles in migraine pathogenesis [12]. They provided evidence that migraine-associated genes were involved in both arterial and smooth muscle function [12]. Meanwhile, as NTG remains a first-line treatment for angina pectoris and acute myocardial infarction, a recent report showed that NTG as a NO donor may be a promising agent to enhance chemotherapy [13], suggesting pleiotropic effects of NTG.

We conclude that patients with migraine without aura in the interictal period have a selective sensitivity in dilator response to NTG and may have systemic NO sensitivity to NTG.

We hope that our study will contribute to the clarification of the pathogenesis and therapy in patients with migraine without aura.
